# Orbital aspergillosis: a case report and review of the literature

**DOI:** 10.1186/s12886-020-01773-7

**Published:** 2021-01-08

**Authors:** Mael Lever, Benjamin Wilde, Roman Pförtner, Cornelius Deuschl, Oliver Witzke, Stefanie Bertram, Anja Eckstein, Peter-Michael Rath

**Affiliations:** 1 Department of Ophthalmology, University Hospital Essen, University Duisburg-Essen, Hufelandstr. 55, 45147 Essen, Germany; 2 Department of Nephrology, University Hospital Essen, University Duisburg-Essen, Essen, Germany; 3 Department of Oral and Maxillofacial Surgery, Kliniken Essen Mitte, University Duisburg-Essen, Essen, Germany; 4 Department of Diagnostic and Interventional Radiology and Neuroradiology, University Hospital Essen, University Duisburg-Essen, Essen, Germany; 5 Department of Infectious Diseases, University Hospital of Essen, University Duisburg-Essen, Essen, Germany; 6 Institute of Pathology, University Hospital of Essen, University Duisburg-Essen, Essen, Germany; 7 Institute of Medical Microbiology, University Hospital Essen, University Duisburg-Essen, Essen, Germany

**Keywords:** Case report, orbital tumour, proptosis, azole resistance, *Aspergillus spp.*

## Abstract

**Background:**

Orbital aspergillosis is a rare sight- and life-threatening fungal infection affecting immunocompromised or otherwise healthy patients. It is often misdiagnosed due to its unspecific clinical and radiologic appearance. Therapeutic delay can have dramatic consequences. However, progress in microbiological diagnostic techniques and therapeutic experience from case series help improve the management of this disease.

**Case presentation:**

A 78-year-old immunocompetent woman presented at an eye clinic for subacute swelling, reddening, and ptosis of her left upper eyelid. Based on radiologic and histologic considerations, she was treated for idiopathic orbital inflammation, but her condition worsened. After a second biopsy of the orbital mass, aspergillosis was diagnosed. Her condition improved promptly after initiation of an oral voriconazole treatment. Additionally, using a polymerase chain reaction (PCR) assay, *A. fumigatus* was identified on tissue of both biopsies and its azole susceptibility was examined simultaneously.

**Conclusions:**

In the case described here, oral antifungal treatment was sufficient for the therapy of invasive orbital aspergillosis. Performing fungal PCR on orbital tissue can accelerate the diagnostic process and should be performed in ambiguous cases of slowly growing orbital mass. Finally, interdisciplinary management is the key to optimal treatment of orbital tumours and infections.

## Background

Orbital aspergillosis is a rare fungal infection mostly presenting as a unilateral orbital mass, which can cause eyelid swelling, proptosis, impairment of ocular motility, and/ or optic nerve compression, leading to vision loss [1]. A further expansion of the mass towards the intracranial cavity due to a delayed diagnosis or inadequate therapy can even be fatal to the patient [2]. Identifying the disease is often complex due to the multitude of possible aetiologies and requires orbital imaging studies as well as histopathological and microbiological examination of a biopsy specimen [3, 4]. In the current literature, described therapeutic approaches vary, ranging from conservative antifungal treatment to radical surgical debulking [5, 6].

Here, we describe the possible obstacles to recognizing this rare disease and report on a case of successful conservative therapy using solely voriconazole. Finally, we present the benefits of using a commercially available polymerase chain reaction (PCR) kit on paraffin-embedded tissue for pathogen identification and simultaneous susceptibility testing.

## Case presentation

**Fig. 1:**
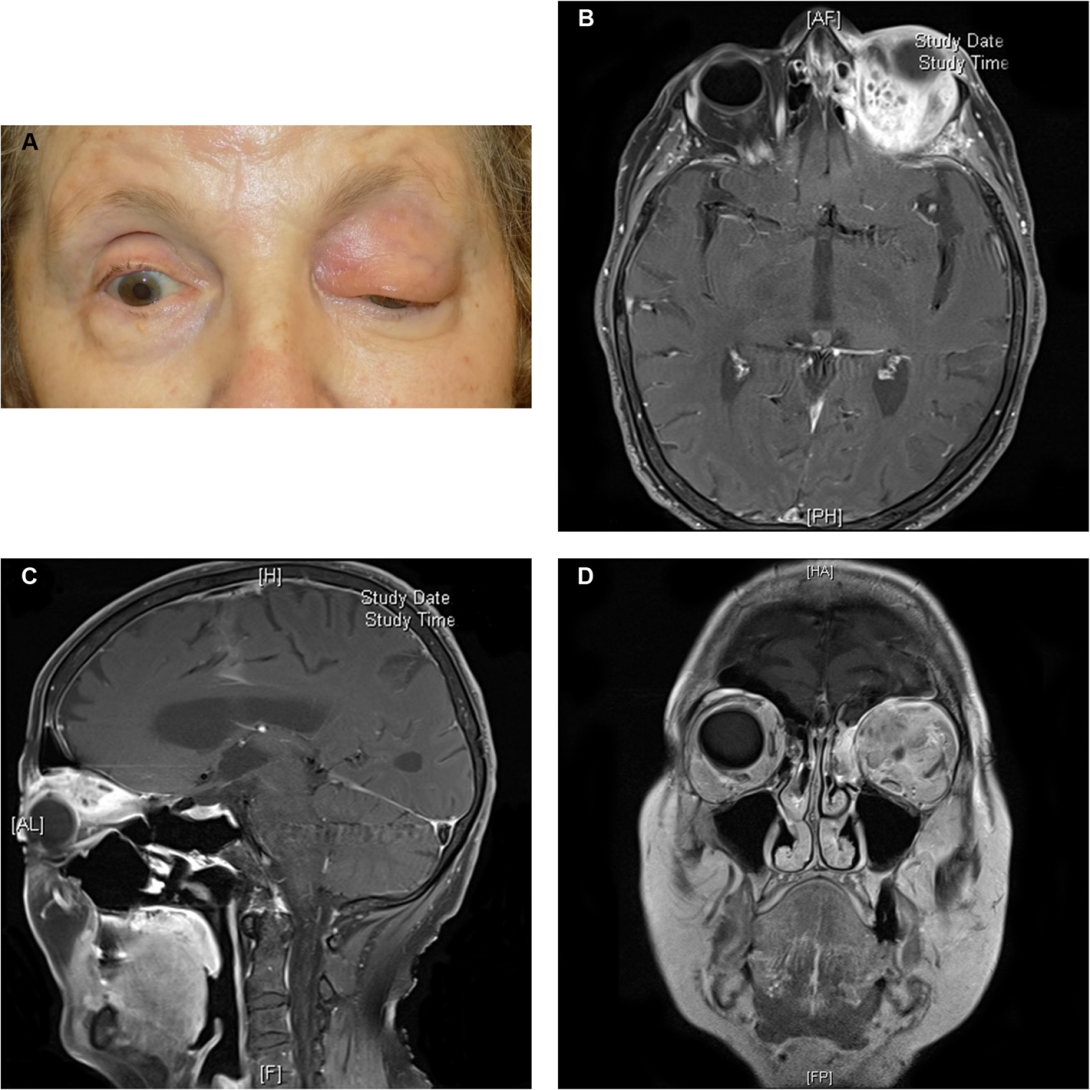
Clinical and radiologic presentation before therapy. **a** shows an en-face photograph of the patient 2 months after the beginning of symptoms. Axial (**b**) and sagittal (**c**) fat-suppressed T1-weighted and coronal (**d**) T1-weighted (without fat suppression) MR images showing the inhomogeneous mass in the upper nasal part of the left orbit

 A 78-year-old woman presented at a tertiary eye clinic due to a painless swelling and reddening of the left upper eyelid and consecutive ptosis for almost two months. Despite a topical treatment with hydrocortisone ointment prescribed 4 weeks previously, the symptoms slowly progressed (Fig. 1). Visual acuity on the left eye was 0.4 (Snellen: 20/50). A slit lamp examination of the left eye showed a conjunctival injection and chemosis, a cataract, and no sign of intraocular inflammation. The optic nerve head and central retina appeared normal. The visual acuity of the right eye was reduced to hand movements due to a complicated retinal detachment treated with vitrectomy and silicone oil tamponade three months previously, but anterior and posterior segment examination showed no abnormalities. However, a proptosis of the left eye and impaired upgaze were observed. The patient appeared in good health apart from arterial hypertension and had no other pre-existing conditions or symptoms, in particular no fatigue, fever or weight loss. Additionally, the patient did not have any indications of immunodeficiency. Vital signs such as arterial blood pressure, heart rate and temperature were normal. The C-reactive peptide concentration and leukocytes count were within normal limits. Anti-neutrophil cytoplasmic antibody (ANCA) and antinuclear antibody (ANA) were not detectable, and immunoglobulin G4 (IgG4) levels were within normal range. An MRI study showed an extraconal mass with an inhomogeneous contrast enhancement expanding to the intraconal compartment. A second MRI after three weeks revealed an additional infiltration of the superior rectus muscle and the ethmoidal bone as well as mucosal thickening and fluid in the ethmoidal sinus (Fig. 1). A biopsy of the mass was acquired using a transpalpebral incision.

**Fig. 2:**
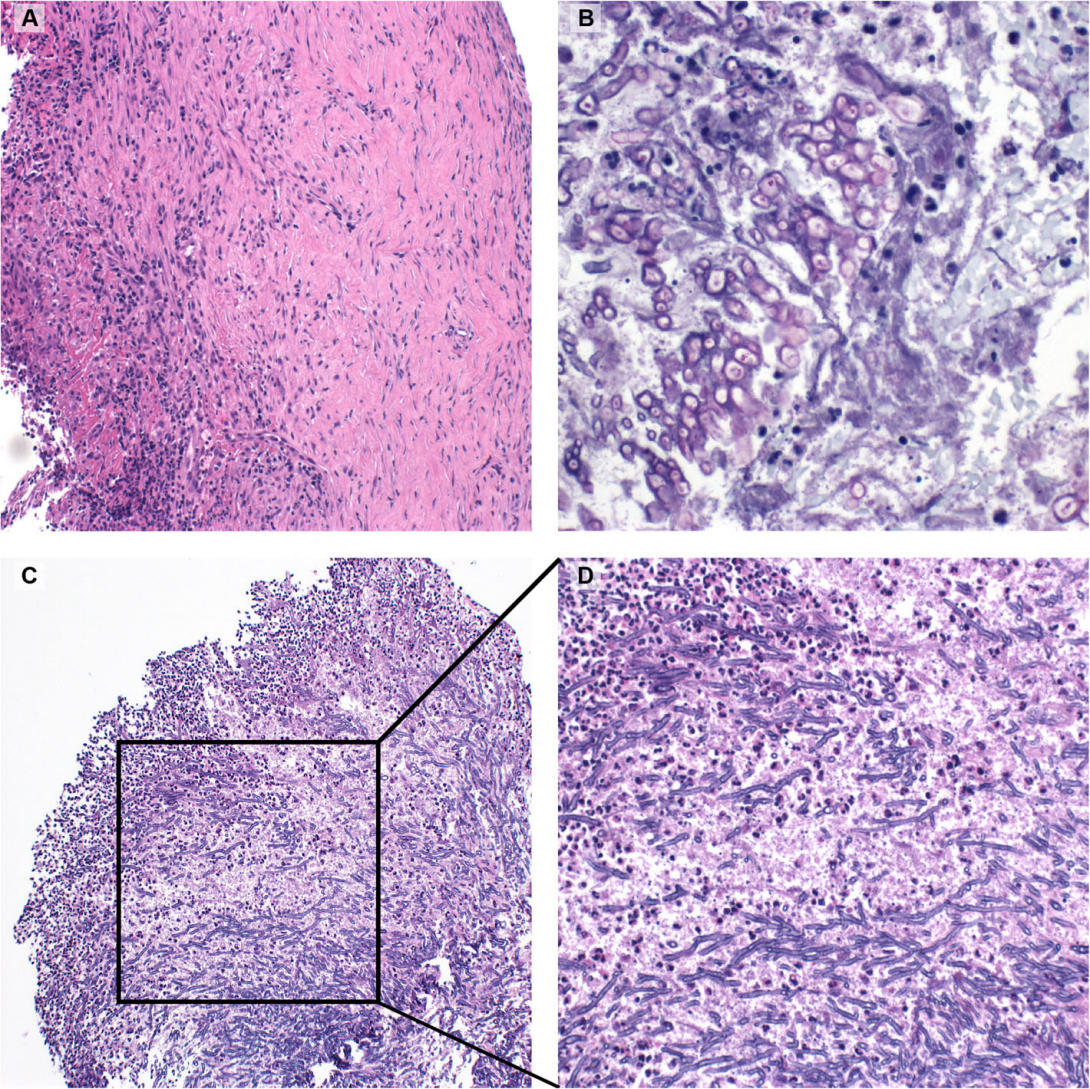
Histopathological aspect of the two biopsy probes. **a** shows a hematoxylin-eosin (HE) stained section of the first biopsy, revealing a mixed lymphocytic and granulocytic inflammation. **b** Periodic acid-Shiff (PAS) staining of the second biopsy showing magenta coloured walls of alive fungi. **c** HE-stained section of the second biopsy showing hyphae with ramification at 45°angle, characteristic for *A. fumigatus*. **d** close caption of the squared region in (**c**)

Macroscopically, the mass appeared inflammatory- or lymphoma-like. In accordance, the histopathologic report showed small areas of unspecific inflammation with lymphocytic, eosinophilic and neutrophilic infiltration (Fig. 2). Consequent immunohistochemic analyses were positive for the B-lymphocyte antigen CD20, as well as for the T-cell marker CD3, and no light chain amplification was observed. The clonality analysis using PCR returned negative. Based on these results and the clinical course of the disease, an idiopathic orbital inflammation (IOI) was diagnosed and a therapy with oral prednisolone was initiated

In the following two weeks under oral prednisolone (80 mg tapered by 10 mg every 4 days), the patient’s condition did not improve. An extended, deeper biopsy specimen was taken, revealing a granulocytic reaction (eosinophilic and neutrophilic) and fungal hyphae with ramification at a 45° angle, characteristic of *Aspergillus spp.*, pointing to the diagnosis of orbital aspergillosis. The patient was transferred to an infectious disease department for further evaluation and therapy. Further blood work continued to show no sign of systemic infection. Blood cultures for bacteria and fungi returned negative, the search for the Aspergillus-antigen galactomannan (GM) was negative (< 0.5), but the (1–3)-β-D-glucan (Fungitell®, Cape Cod, MA, USA) serology was positive (113 pg/mL), thus accounting for an invasive fungal infection. Given these results, an oral therapy with voriconazole was initiated. In the following days, the plasma trough concentrations of voriconazole were within the target range of 1.0–5.5 mg/L.

 Using PCR (AsperGenius kit, PathoNostics, Maastricht, The Netherlands) on paraffin-fixed biopsy tissue, *A. fumigatus* was identified in both biopsy specimens. Using the same test, mutations that are typically associated with azole-resistance were ruled out.

**Fig. 3:**
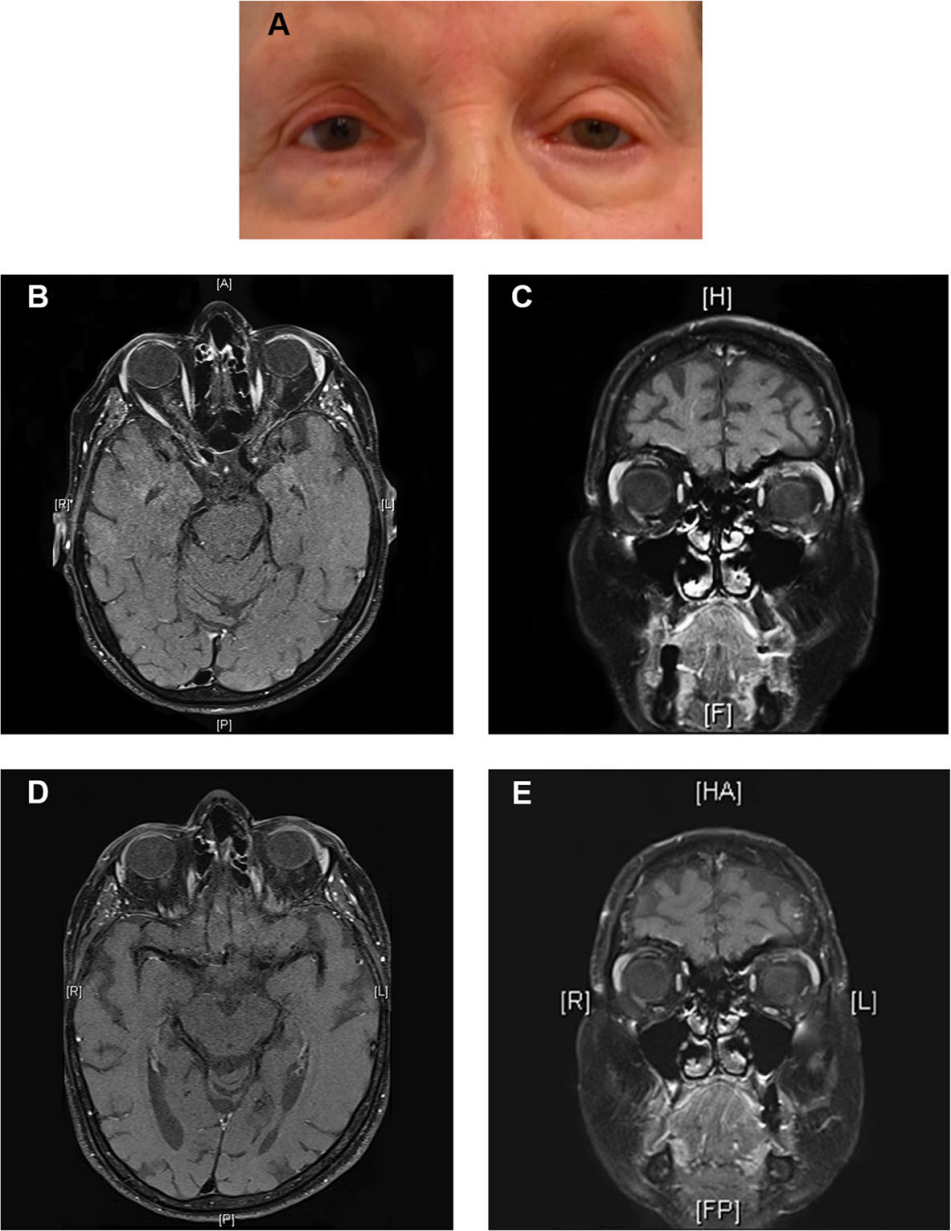
Clinical and radiologic presentation after initiation of antifungal therapy. **a** shows an en-face photograph of the patient 14 weeks after beginning the antifungal therapy. Axial (**b**, **d**) and sagittal (**c**, **e**) fat-suppressed T1-weighted and coronal (**d**) T1-weighted (without fat suppression) MR images showing an almost complete regression of the mass in the left orbit 11 months (**b**, **c**) and a complete regression of the mass 24 months (**d**, **e**) after the beginning of the therapy

In the following 2 years, under sustained oral voriconazole treatment, the patient showed a clear clinical and radiologic improvement (Fig. 3), and (1–3)-β-D-glucan turned negative after 3 months. The best-corrected visual acuity (BCVA) for the left eye recovered to 0.7 (Snellen: 20/25). Since the therapy was well tolerated and the drug concentration stayed within therapeutic range, the voriconazole treatment was continued to minimize the risk of relapse.

## Discussion and conclusion

This case report is interesting for three main reasons: (1) even using state of the art methods, orbital inflammation can be diagnostically challenging, (2) oral antifungal drugs may be an alternative to radical surgery for the treatment of selected cases of orbital aspergillosis, and (3) PCR can accelerate the initiation of the correct therapy by identifying *Aspergillus spp.* and testing for possible resistance.

*Aspergillus spp.* are ubiquitous saprophytes responsible for a rising number of infections in humans. *A. fumigatus* is the most common human pathogen, colonizing the upper airways from where it can spread to the paranasal sinuses. Life-threatening invasive forms of aspergillosis are observed among the growing population of immunocompromised patients. Surprisingly, *A. fumigatus* infections of the orbit are more commonly reported, like in our case, in immunocompetent patients [4]. Even though it is considered as rare, the prevalence of orbital aspergillosis is growing [3]. The high mortality rate among patients with aspergillosis is caused by the infection spreading from the orbit towards the intracranial cavity via the orbital fissures or the optic canal [1]. Timely diagnosis and prompt initiation of an adequate antifungal therapy is of great prognostic value as misdiagnosis and initial treatment with high-dose corticosteroids is associated with a more probable recurrence of the infection and fatal outcome [4, 7].

One cause of diagnostic delay is the clinical similarity of orbital pathologies often presenting with unspecific symptoms like periorbital swelling, proptosis, diplopia and impaired eye movement, ptosis, visual impairment, and conjunctival reaction (inflammatory or due to restricted venous drainage). The most relevant differential diagnoses for aspergillosis are vascular (carotid cavernous fistula), specific inflammatory (sarcoidosis, granulomatosis with polyangiitis, IgG4-associated disease, Erdheim-Chester disease) or non-specific (idiopathic orbital inflammation [IOI]), neoplastic (lymphoma, metastasis, etc.), or infectious (bacterial, fungal). Finally, orbital processes can appear secondary to an infection of the neighbouring paranasal sinuses.

Radiologic studies are useful for the elucidation of orbital conditions, providing important information about size, origin, and expansion of the mass or infiltration into essential neighbouring structures such as blood vessels, extraocular muscles or the optic nerve. However, their availability depends greatly on local health care systems. In orbital aspergillosis, computed tomography can be helpful as it can reveal calcifications within the mass, which are almost pathognomonic for aspergillosis, and it allows for a precise evaluation of bony structure involvement. In MRI, aspergillosis appears as an isointense lesion in T1 with intense contrast enhancement after injection of gadolinium and the hypointense appearance on T2-weighted sequences, but these signs are rather unspecific [8]. In our case, the two orbital MRI studies made a lymphoma or other lesion like IOI the most probable diagnoses. Still, a subacute sinugenic infection could not be ruled out, in particular because of the accompanying ethmoid sinusitis. Radiologic studies are thus insufficient for definitive diagnostic clarification, which makes a histopathological examination of the mass crucial.

Orbital connective tissue is highly reactive, as observable in the first biopsy. The mixed inflammatory infiltration there corresponds to the reaction expected around an infection. Fungal hyphae branching at 45° angle are characteristic of aspergillosis. These are best visible using periodic acid-Shiff (PAS) or Gomori methanamine silver stainings [9]. However, due to inappropriate staining method and/or sampling error, the sensitivity of microscopy is low (between 33 and 50%) and, consequently, the need for repeated biopsies – like in our case – is frequently reported [3]. Whenever an infection is suspected, additional tissue should be obtained for microbiological culturing [10]. In the case of invasive aspergillosis, serologic tests are also available: detection of the antigen galactomannan (GM) via enzyme-linked immunosorbent assay (ELISA) is highly sensitive [10] and (1–3)-β-D-glucan is also often positive in invasive fungal infections, but the reliability of these methods can be affected by false positive results [7, 10]. Alternatively, PCR provides a high sensitivity and specificity for *Aspergillus* species including *A. fumigatus* [11].

The therapy of orbital aspergillosis has evolved significantly over the past decades. Initially, radical surgical resection of affected tissue and, if necessary, orbital exenteration was considered the therapy of choice. This concept is now considered obsolete as, in invasive infections, it is difficult to determine the precise extent of the disease, and the resection of vital structures like bone, blood vessels or nerves has dramatic consequences [5]. Moreover, surgery guaranties neither a successful control of the disease[4] nor a survival of the patient [12]. In the last decades, reports of satisfying therapeutic results using combinations of less radical surgery and systemic antifungal therapy or even conservative treatment regimens have been published [1, 12]. Antifungal therapy can consist of polyenes (amphotericin B), azoles (e.g., voriconazole, posaconazole), or echinocandins (e.g., caspofungin). Information on the efficacy of antifungal treatments for orbital aspergillosis is based solely on small case series, in which various drugs were used in addition to surgery [1, 13]; in these cases, survival rate was high (one death out of 14 patients in the report by Pushker et al. [1]). Other patients were treated with either amphotericin B or its combination with itraconazole[12, 14] also with encouraging results. While voriconazole was shown effective and well tolerated [6], the present case represents the fifth where it was used alone for treatment of orbital aspergillosis [15–18]; in these few cases, as in ours, the therapy was successful. Amphotericin B is decreasingly relevant due to frequent reports of nephrotoxicity [6]. In contrast, voriconazole showed a higher efficacy, lower toxicity, and its intravenous and oral availability allows for more flexibility during months-long therapies. It is now the first line treatment for invasive pulmonary aspergillosis [10]. Though one should be careful to not generalize based on a limited number of cases, reports of successful conservative therapy advocate for a broader indication of medical treatment in sino-orbital aspergillosis, as opposed to radical surgery. This positive experience with oral voriconazole should motivate further studies on its therapeutic potency.

Regrettably, the positive experience with azoles for the treatment of invasive orbital aspergillosis must be relativised given the rise of resistant *Aspergillus* strains [19–21]. Azole resistance is of high clinical relevance as it is the cause of fatal treatment failures. Accordingly, the European Society for Clinical Microbiology and Infectious Diseases recommends performing susceptibility testing in regions with known resistances [7]. Resistance against azoles primarily involves mutations in the CYP51A gene [22]. In addition to microbiological culture, commercial PCR kits are available to identify *Aspergillus spp.* directly from clinical samples and search simultaneously for genetic mutations that confer resistance to azoles. PCR was also shown to be more sensitive and specific than serologic methods for detecting aspergillosis [11] and results are usually available within hours, thus potentially accelerating the initiation of the adequate therapy. Our case is the first to use PCR on paraffin-fixed periocular tissue successfully. Here, the method was able to identify *A. fumigatus* as the cause of a sino-orbital infection and to exclude with high probability an azole resistance. Interestingly, the AsperGenius PCR kit was also positive using tissue from the first biopsy, which was histopathologically inconclusive. Our experience suggests that a PCR analysis can be relevant in ambiguous, histologically negative cases, particularly since it can be performed retrospectively as a two-step procedure: first, a universal fungal PCR followed by an *A. fumigatus* specific resistance PCR, if indicated. This diagnostic approach should be tested in larger cohorts.

In conclusion, orbital aspergillosis is a sight- and life-threatening condition. It should be considered in the case of a slowly growing orbital mass. While radiologic studies are informative, definitive diagnosis requires the pathological and microbiological analysis of an orbital biopsy. The present case suggests that medical treatment according to susceptibility testing can be sufficient even in invasive disease. Genotypic characterization of *A. fumigatus* and antifungal resistance testing can accelerate the initiation of therapy and may reduce the need to repeat biopsy. Finally, facing the difficulties of diagnosis and therapy of orbital aspergillosis, an interdisciplinary approach between ophthalmologists, rhinologists, maxillo-facial surgeons, pathologists, microbiologists and infectious disease experts is the key to optimal disease management.

## Data Availability

The datasets used and/or analysed during the current study are available from the corresponding author on reasonable request.

## Abbreviations

GM: Galactomanan
IOI: Idiopathic orbital inflammation, formerly pseudotumor orbitae
MRI: Magnetic resonance imaging
PAS: Periodic acid-Shiff staining
PCR: Polymerase chain reaction
